# MicroRNAs as a Tool for Differential Diagnosis of Neuromuscular Disorders

**DOI:** 10.1007/s12017-023-08763-0

**Published:** 2023-10-19

**Authors:** Nahla O. Mousa, Ahmed Abdellatif, Nagia Fahmy, Hassan El-Fawal, Ahmed Osman

**Affiliations:** 1 Biotechnology Department, Faculty of Science, Cairo University, Giza, 12613 Egypt; 2 Biology Department, School of Sciences and Engineering, The American University in Cairo, Cairo, 11835 Egypt; 3 Neuropsychiatry Department, Faculty of Medicine, Ain Shams University, Cairo, 11566 Egypt; 4 Institute of Global Health and Human Ecology, School of Sciences and Engineering, The American University in Cairo, Cairo, 11835 Egypt; 5 Biotechnology Department, Basic and Applied Sciences Institute, Egypt-Japan University of Science and Technology, Borg Al Arab, 21934 Egypt; 6 Biochemistry Department, Faculty of Science, Ain Shams University, Cairo, 11566 Egypt

**Keywords:** Differential diagnosis, Muscular dystrophy, MicroRNAs, miR-499

## Abstract

Neuromuscular disorders (NMD) are a class of progressive disorders that are characterized by wasting of the muscles. Some of the disorders like Duchenne muscular dystrophy (DMD), Becker muscular dystrophy (BMD), congenital muscular dystrophies (CMDs), limb-girdle muscular dystrophies (LGMD), and mild spinal muscular atrophy (SMA) type III share several presenting clinical features, and hence, diagnosis is usually a challenging task. In this study, the diagnostic potential of some species of microRNAs (miRNAs) that are known to play roles in normal and pathological contexts of myocytes (myomiRs) were evaluated to assess their potential in differential diagnosis of NMDs. In this study, seventy-four patients with different neuromuscular disorders along with thirty age-matched healthy control subjects were enrolled. Peripheral blood samples were collected from enrolled subjects followed by miRNA extraction and reverse transcription followed by quantification of the circulating levels of the studied miRNAs (miR-499, miR-206, miR-208a, miR-223, miR-191, miR-103a-3p, miR-103a-5p), by real-time PCR and statistical analysis. The data indicated that miR-499 level showed high circulating levels in DMD patients as well as in patients with other related disorders such as BMD. However, the levels of miR-499 were much higher in DMD patients and it can be used to diagnose DMD. In addition, miR-206 can selectively differentiate between DMD and all other disorders. The results also revealed that miR-208a and miR-223 were significantly dysregulated in SMA patients, and miR-103a-3p could distinguish DMD from BMD. The expression levels of some miRNA species can be utilized in the process of differential diagnosis of NMDs and can serve as a diagnostic biomarker, and such findings will pave the way towards generating targeted therapies.

**Supplementary Information:**

The online version contains supplementary material available at 10.1007/s12017-023-08763-0.

## Introduction

Muscular dystrophies (MDs) are a class of inherited neuromuscular disorders (NMDs) that occur due to mutation in muscle-related genes that consequently leads to abnormal expression of muscular proteins (McNally & Pytel, 2007). According to the gene affected, MDs can be subdivided into different forms like Duchenne muscular dystrophy (DMD); Becker (BMD); Myotonic dystrophy (DM); and limb-girdle (LGMD) which is considered the most heterogenous type of muscular dystrophies. In MDs, the patients’ cardiac muscle is affected even if they are asymptomatic and they usually die due to pulmonary and cardiac failure (Lidov, 2000).

The most fatal form of muscular dystrophy is Duchenne muscular dystrophy which occurs due to aberration in the DMD gene-encoding muscular dystrophin protein. Due to the recessive X-chromosome linked nature of the disease, it affects mainly boys in their early childhood and usually causes death in adolescence (Werneck et al., 2019). The disease occurs due to a break down in the sarcolemma, due to the absence of functional dystrophin protein, which gradually lead to muscle wasting and accumulation of matrix proteins (Nowak & Davies, 2004). When DMD is suspected from the observed clinical picture, additional investigations are usually requested like measuring creatine-kinase (CK) levels in blood (Zatz et al., 1991) and identifying abnormal protein expression in muscle biopsy (Barresi, 2011).

However, several disorders can lead to a phenotype like that of DMD and should be considered in the differential diagnosis especially if they are potentially treatable. Becker Muscular Dystrophy resembles DMD in the etiology of the disease; however, it is milder due to the presence of mutated, semi-functional dystrophin protein (Freund et al., 2007). Another group of genetic myogenic diseases is Limb-girdle muscular dystrophy (Wang et al., 2018), a non-congenital form of muscular dystrophies. LGMD comprises two main subtypes based on the pattern of inheritance: the autosomal recessive (LGMD-AR) and the autosomal dominant (LGMD-AD), and each subtypes include many other forms according to the genetic cause of the disease that affects the production of muscular proteins in the sarcolemma and sarcoplasm in addition to other parts of muscle fiber like Desmin, Calpain-3, Dysferlin, Sarcoglycan, Titin, etc. Mutations in the genes encoding these proteins lead to wasting in the muscles of proximal limb, and cardiomyopathy is always associated with the disease. High-throughput sequencing usually discovers almost all LGMD-associated mutations, which facilitate understanding the underlying mechanism of disease pathogenesis as a result of the expression of inactive proteins due to structural and/or functional impairment (Bushby et al., 2007).

Another class of neuromuscular diseases is motor neuron diseases like spinal muscular atrophy (SMA) (Kolb & Kissel, 2015) that affects motor nerves in the spinal cord and consequently lead to progressive skeletal muscle atrophy. SMA usually affects the proximal muscles before distal muscles. SMA can occur in 5 different patterns due to mutations in spinal motor neuron-1 (*SMN1*) gene, which lead to the production of a truncated SMN protein, an event that adversely affects the process of brain-muscle signaling and consequently leads to skeletal muscular atrophy. In some instances, the expression of a partially functional SMN protein from *SMN2* gene may reduce the severity of the disease and ameliorate the symptoms. The prevalence of SMA reaches about 1 child in every 10,000 children. The clinical features of SMA include proximal muscle weakness, and it extends to reach bulbar musculature and intercostal muscles (D'Amico et al., 2011). In addition to MDs and SMA, Congenital myopathies are a group of diseases that disrupt the muscular tissue and appear as proximal or general static muscle weakness and hypotonia with dysmorphic myofibers structure, and it is usually accompanied with pulmonary insufficiency (Ravenscroft, et al., 2018). Myopathies are wide class of NMDs that include inherited disorders (like muscular dystrophies) as well as acquired disorders (Cassandrini et al., 2017). GNE myopathy (Nonaka distal myopathy with rimmed vacuoles) is one of the myopathies that resulted from a mutation in GNE gene which encodes for N-acetylglucosamine 2-epimerase/N-acetylmannosamine kinase which is crucial in the biosynthesis of N-acetylneuraminic acid (sialic acid) (Awasthi et al., 2019). Its prevalence is 1/1.000.000 which classify the disease as an “ultra-rare” disease. The symptoms of the disease usually appear in the third decade, and the distal muscle weakness starts in the legs of the TA muscle and then reaches the muscles of the upper limbs. The disease is characterized by rimmed vacuoles and lamellar (myeloid) body depositions and accompanied by mild elevation in serum CK (Pogoryelova et al., 2018). Collagen VI-related myopathies are a class of myopathies that result from genetic mutations in the genes expressing α-chains of collagen VI. The characteristic common feature of these diseases is the disruption of the muscular tissue in addition to the connective tissue (Kim et al., 2018).

Diagnosis of NMDs mainly depends on clinical pictures, obtaining the patients’ family history, and assessing the musculoskeletal and neurological functions (Birnkrant et al., 2018). For the inherited NMDs, different types of DNA testing are usually performed to detect the exact genetic aberration. However, despite the continuous updates in the list of disease-associated mutations, some mutations may escape detection, and new ones may appear and not searched for, and hence, some patients may remain undiagnosed (Splinter et al., 2018). Therefore, finding accurate, robust, and versatile diagnostic/prognostic biomarkers is considered a hot research topic, not only for NMDs, but also for almost all diseases.

Muscle-specific miRNAs (myomiRs) are specific species of miRNAs that are mostly expressed in the muscles and play significant roles in the development of muscular system and its regeneration and differentiation (Sjogren et al., 2017). MyomiRs can be used as auspicious markers for identifying the underlying muscular disorder (Huang, 2017). For orphan neuromuscular diseases, few research studies have been carried out aiming for developing new biomarkers. Koutsoulidou and co-workers (REF) highlighted the significance of microRNAs as circulating biomarkers, and they showed that miR-223-3p and miR-206 are promising circulating biomarkers for Facioscapulohumeral Muscular Dystrophy type 1, miR-143-3p, and miR-486-3p for Limb-Girdle Muscular Dystrophy type 2A whereas miR-363-3p and miR-25-3p associate with Myotonic Dystrophy type 2 (Koutsoulidou et al., 2022).

Moreover, miR-1, miR-133a/b, miR-206, miR-208a/b, and miR-378a were also found to be dysregulated in serum samples of Duchenne Muscular Dystrophy, Becker Muscular Dystrophy, Facioscapulohumeral muscular dystrophy, Myotonic Dystrophy type 1, and Myotonic Dystrophy type 2 patients (Hu et al., 2014; Li et al., 2014; Matsuzaka et al., 2014; Perbellini et al., 2011; Zaharieva et al., 2013).

In this study, we evaluated the diagnostic applications of some myomiRs, aiming for identifying a non-invasive diagnostic method, and assessed their potential in differential diagnosis in patients with different types of NMDs and similar presented clinical pictures and disease phenotype. We have determined the circulatory levels of the selected miRNAs in different forms of Muscular Dystrophy with emphasis on DMD, BMD, LGMD, myopathies, and SMA.

## Materials and Methods

### Study Participants and Sample Collection

One hundred and four subjects were enrolled in the current study. The subjects were categorized into 2 main cohorts: NMDs cohort includes 74 patients (18 Duchenne Muscular Dystrophy, 5 Becker Muscular Dystrophy,18 Limb-girdle Muscular Dystrophy, 10 Congenital Muscular Dystrophy, 15 Congenital myopathy and 8 Spinal muscular atrophy) and control cohort includes thirty ages matched healthy subjects; 15 males and 15 females with age ranging from 20 to 30 years. All the patients were clinically examined prior to enrollment in the study. The patients were diagnosed to have the respective disease along with identifying disease stage using clinical and appropriate laboratory investigations. None of the patients suffered from any other disease that might affect the circulating miRNA profile or levels. The control subjects did not have any family history for any muscle disorder, nor did they have any malignancy or inflammatory disease.

The inclusion criteria for including boys with DMD or BMD in the study were as follows: they are Genetically proven to be DMD or BMD using MLPA and NGS which are used to detect dystrophin gene mutations. The age range of the DMD boys was from 1.5 to 14 years, and for the BMD, the range was from 13 to 30 years. Muscle strength was evaluated along with the cardiac function.

For the SMA group, samples were collected from eight male subjects who suffered from neurogenic SMA type I or type III according to confirmed genetic diagnosis. The age range of the SMA participants was from 3 to 29 years.

For the LGMD group, 18 patients were participated including 10 males and eight females aged from 14 to 38 years with a Clinical phenotype of titinopathy, calpainopathy, sarcoglycanopathy, and dysferlinopathy. Blood DNA was used for genetic analysis, and muscle biopsies were also investigated.

For the congenital muscular dystrophy group, the group included five boys and five girls; four of which had LAMA-2-related Muscular Dystrophy (age range from 11 to 18 years). Four patients had congenital myotonia (age range from 4 to 12 years) and two patients with congenital muscular dystrophy (6 years girl and 13 years boy).

The last group of patients included 15 patients; 4 males had Collagen VI-related myopathy (with age range from 27 to 40 years), 4 males had GNE myopathy (with age range from 21 to 40 years), and 7 patients with congenital myopathy (4 girls and 3 boys with age range from 1 to 5 years).

Venous blood samples (2 ml, on EDTA-vacutainers) were collected from all study subjects. Plasma samples were subsequently separated, in less than 4 h from the time of collection and kept frozen until used for analyzing the underlying circulating biomarkers.

### miRNA Extraction and Reverse Transcription

In this study, the expression levels of 7 microRNA species (miR-499, miR-206, miR-208a, miR-103a-3p, miR-103a-5p, miR-191, and miR-223) were carried out to investigate their potential as biomarkers for differential diagnosis in NMDs. The selection of the tested miRNAs was based on their involvement in the regulatory processes in myocytes, which was drawn from the literature survey. The selection was further confirmed by searching specific databases such as miRSearch, v. 3.0, miRBase, and TargetScan v. 7.2 to identify target genes, which proved involvement in normal and pathological contexts of muscle cells.

Circulating microRNA was extracted from 200 μl of plasma using the miRNeasy Serum/Plasma Advanced kit (Qiagen, Germany), following the vendor’s recommended instructions. A synthetic Caenorhabditis elegans miRNA-39 (Cel-miR-39; 5′-UCACCGGGUGUAAAUCAGCUUG-3′; Qiagen, Germany) was spiked into each serum sample followed by miRNA extraction using the regular protocol. Total circulating miRNA was resuspended in 20 μl of RNase-free water, and sample concentration and purity were evaluated using a Nanodrop™ 2000c spectrophotometer (ThermoFisher Scientific, USA).

Reverse transcription reactions were assembled using 100 ng (in microliter equivalent) of the extracted miRNA in a 10 μl reactions using the miScript II RT kit (Qiagen, Germany) according to the manufacturer’s recommendations.

### Quantitative Real-Time PCR

To determine the circulating levels of the selected miRNAs, we used miScript SYBR Green PCR kit (Qiagen, Germany), using miRNA-specific primers along with an aliquot of each RT reaction and the kit’s 2X master mix and following the manufacturer’s instructions. All reactions were carried out in triplicates.

PCR was performed for 15 s at 95 °C; 30 s at 55 °C, and 30 s at 70 °C for 40 cycles and finalized by a high-resolution melt analysis with 5-s intervals for each 0.5 °C. The CFX Connect Real-time PCR System (Bio-Rad, USA) was used for real-time PCR. Finally, the samples were analyzed using CFX Manger (Bio-Rad). All miRNA expression levels were normalized to the spike-in control, cel-miR-39 expression.

### Statistical Analysis

Statistical analysis was performed using IBM SPSS® Statistics version 23 (IBM® Corp., Armonk, NY, USA). The Mann–Whitney test (non-parametric t-test) was used for quantitative data that are not normally distributed. The Spearman-rho method was used to test correlation between numerical variables. Wilcoxon signed ranks test (non-parametric paired *t*-test) was used for correlation analysis. The Receiver Operating Characteristic (ROC) curve was used for prediction of cut off values for differentiating patients form the control healthy persons and presented as area under the curve and its 95% confidence interval. All tests were 2 tailed. A *p* value at α < 0.05 is considered significant. No missing values were reported.

## Results

In our study, we investigated the potential of some species of circulating miRNAs to differentiate between the most common forms of neuromuscular disorders, and in particular, the ability of such serum-circulating levels and dysregulation to differentiate between patients with Duchenne muscular dystrophy and other forms of muscular dystrophies like Becker muscular dystrophy, limb-girdle muscular dystrophy, and congenital muscular dystrophies.

**Fig. 1:**
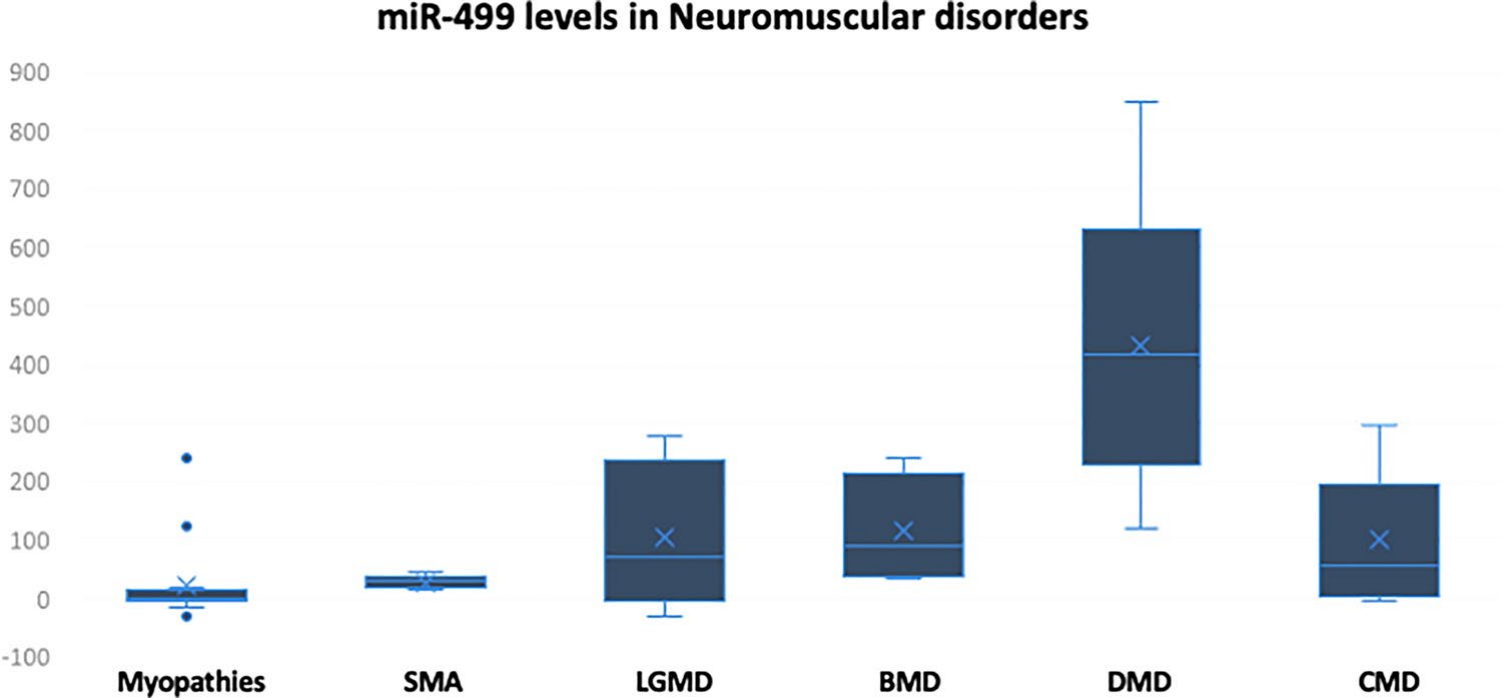
Expression levels of miR-499 represented in RQ values in different neuromuscular disorders compared to the control group

To investigate the dysregulation of the selected microRNA species across different disorders, real-time PCR was carried out, and Livak analysis was performed. Regarding miRNA-499, the circulating levels of this species were found to be upregulated in 100% of the DMD, BMD, and congenital muscular dystrophy patients. In fact, miR-499 was not expressed in the controls and the Ct cycles in all the reactions from the control subjects were 40 or above. The RQ values in case of the DMD patients (mean RQ = 418.15) were much higher than in BMD (mean RQ = 98.7), LGMD (mean RQ = 144.7), and Congenital MD as well (mean RQ = 83.13) **(**Fig. 1 and Table 1). Patients with myopathies also showed upregulation in the expression of mir-499, but the levels (mean RQ = 22) were significantly lower when compared to that of other NMDs. In addition, patients with SMA had upregulated levels of miR-499 compared to the healthy group (mean RQ = 102.81). Thus, miR-499 levels in DMD >  > LGMD > SMA > BMD > Congenital MDs > Myopathies. Furthermore, by comparing the levels between the different groups, it was clear that miR-499 has the potential to differentiate between the control group and all the tested disorders, and it can even be utilized to differentiate between DMD and other MDs, when the serum-circulating levels (RQ values) are taken in consideration.

**Table 1:** Descriptive analysis showing the frequency of cases with increased or decreased levels of miR-499 across different disorders:

Disease /miR-499 levels	Total no	RQ > 1.5	RQ < 0.9	RQ 0.9–1.5
Myopathies	15	6	9	0
Spinal muscular atrophy	8	8	0	0
Limb-girdle muscular dystrophy	18	11	7	0
Becker muscular dystrophy	5	5	0	0
Duchenne muscular dystrophy	18	18	0	0
Congenital MD	10	9	1	0

**Fig. 2:**
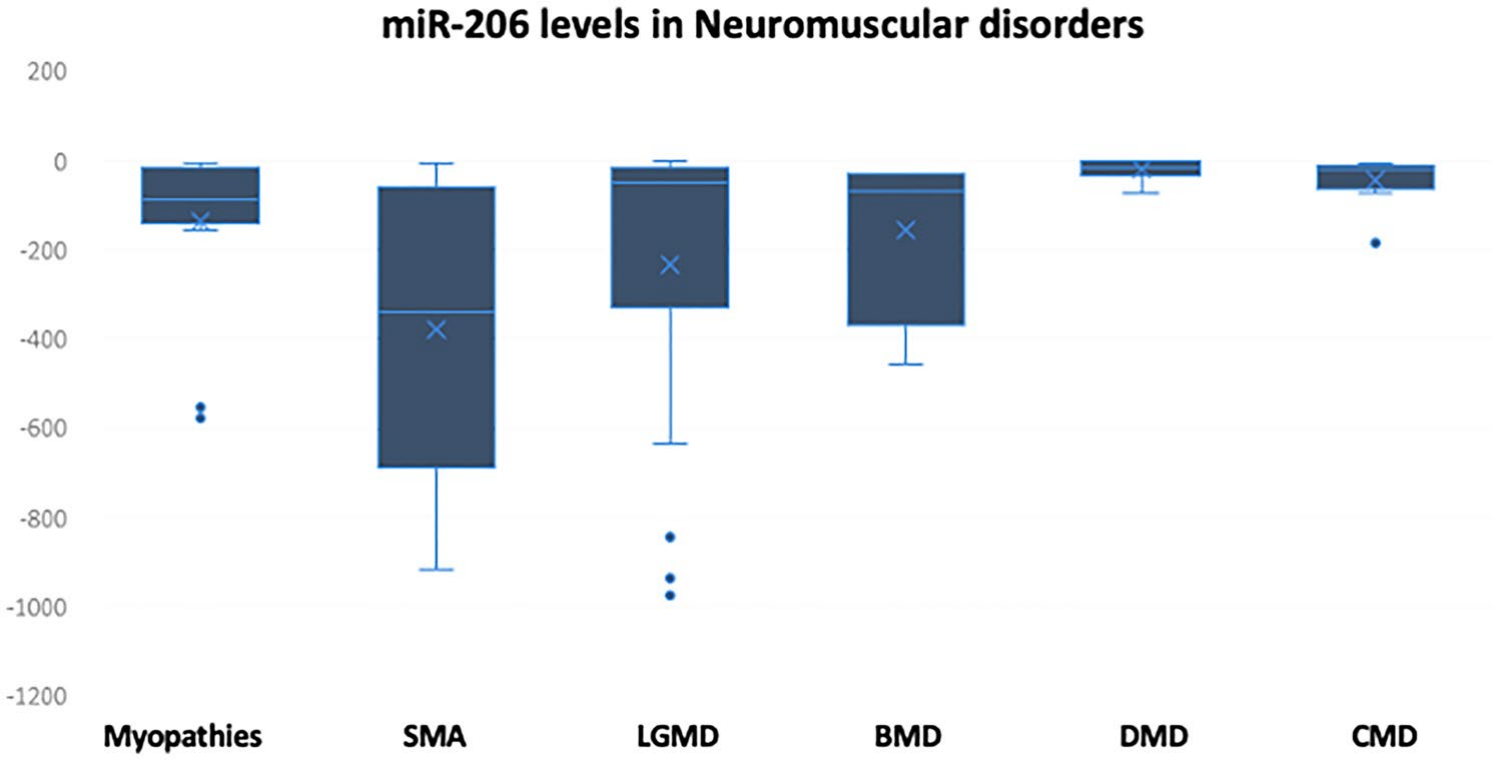
Expression levels of miR-206 represented in RQ values in different neuromuscular disorders compared to the control group

Next, we assessed the circulating levels of miR-206 in the patients’ sera and compared the results to that of the control group. Interestingly, all the patients with Spinal Muscular Atrophy (100%) exhibited significant downregulation for this miRNA species with extreme low levels of miR-206 (mean RQ  = − 1222.4). In addition, all the patients with congenital MD (100%) showed mild reduction in the circulating levels of miR-206 (mean RQ = − 40.84) as well as 17 out of the 18 patients with LGMD (94.44%) had relatively low levels (mean RQ = − 427.19). In case of DMD, 15 patients out of a total of 18 patients (83.33%) enrolled in the study showed slight downregulation in miR-206 levels (mean RQ = − 21.3) while the other 3 DMD patients had control-like levels. Patients with different myopathies also show low levels of miR-206 (mean RQ = -135.2) (Fig. 2 and Table 2). The downregulation of miR-206 in case of SMA >  > LGMD >  > Myopathy > BMD > Congenital MD > DMD. To determine the potential of miR-206 to be used as a biomarker to differentiate between the healthy subjects and the NMD patients or to differentiate between different disorders, Kruskal–Wallis’ analysis was carried out to compare the mean values of different groups, and our results revealed that miR-206 can differentiate between controls and all NMD patients. In addition, miR-206 could also differentiate between SMA and DMD (*p* < 0.007) as well as between DMD and congenital MDs (*p* = 0.028).

**Table 2:** Descriptive analysis showing the frequency of cases with increased or decreased levels of miR-206 across different disorders:

Disease & miR-206 levels	Total no	RQ > 1.5	RQ < 0.9	RQ 0.9–1.5
Myopathies	15	0	15	0
Spinal muscular atrophy	8	0	8	0
Limb-girdle muscular dystrophy	18	0	17	1
Becker muscular dystrophy	5	0	5	0
Duchenne muscular dystrophy	18	0	15	3
Congenital MD	10	0	10	0

**Fig. 3:**
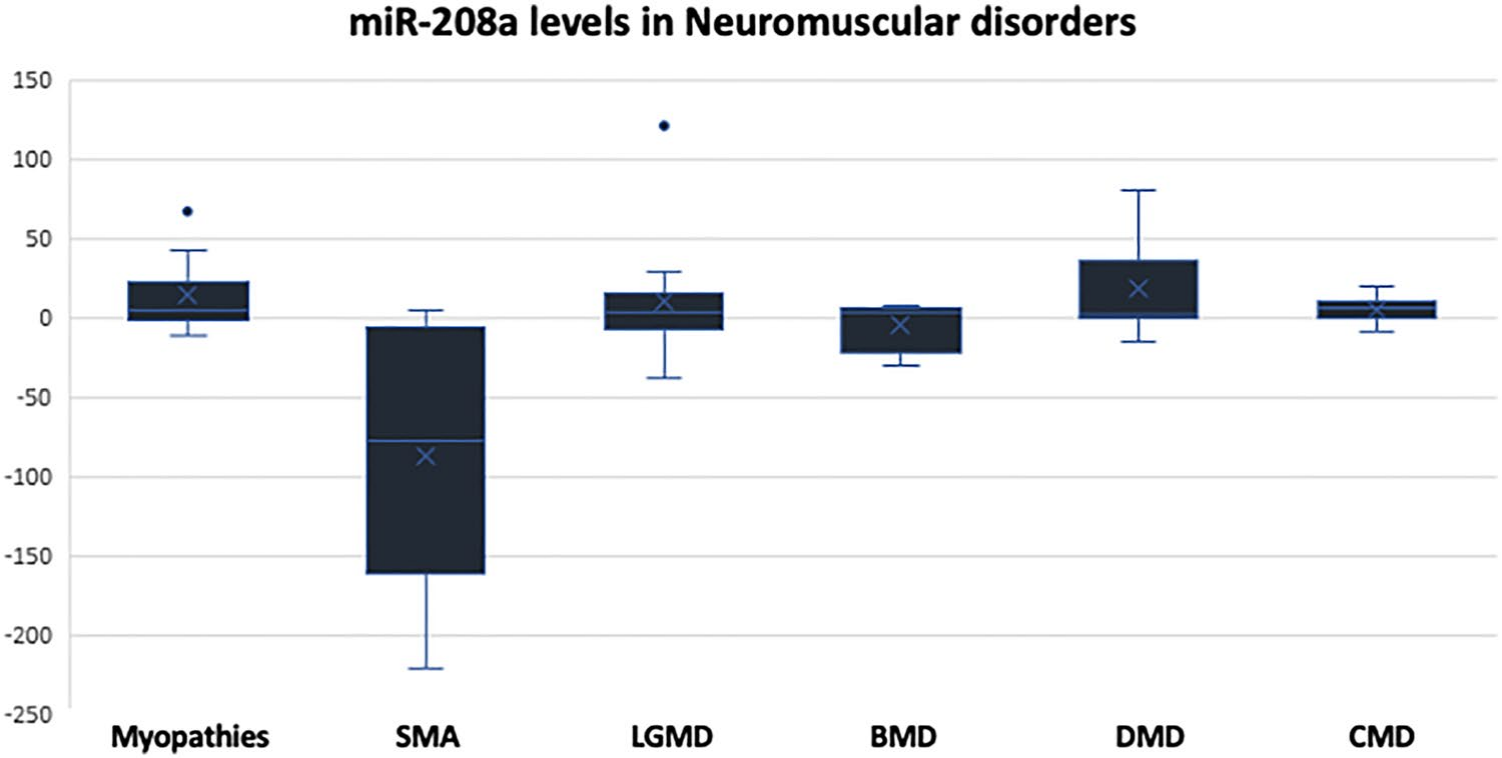
Expression levels of miR-208a represented in RQ values in different neuromuscular disorders compared to the control group

We further evaluated the levels of miR-208a in the sera of the patients and the control as well. Spinal muscular atrophy patients had the lowest levels of miR-208a (mean RQ = − 228.73). On the other hand, 73.3% of the patients with myopathies (11 patients out of 15), 61.1% of the Limb-Girdle MD (11 patients out of 18), 61.1% of DMD (11 patients out of 18), and 40% of the BMD (2 patients out of 5) showed high levels of miR-208a while the rest of the patients showed levels comparable to that of the control group or decreased levels of miR-208a. The mean RQ values of miR-208a in SMA < LGMD < BMD < Congenital MDs < Myopathies < DMD **(**Fig. 3 and Table 3). Kruskal–Wallis’ analysis revealed that miR-208a can be used as a potent biomarker to differentiate SMA from other NMDs since it exhibited significantly low levels in all SMA patients, and the circulating levels were much downregulated than that of other tested NMDs.

**Table 3:** Descriptive analysis showing the frequency of cases with increased or decreased levels of miR-208a across different disorders:

Disease & miR-208a levels	Total no	RQ > 1.5	RQ < 0.9	RQ 0.9–1.5
Myopathies	15	11	4	0
Spinal muscular atrophy	8	1	6	1
Limb-girdle muscular dystrophy	18	11	7	0
Becker muscular dystrophy	5	2	1	2
Duchenne muscular dystrophy	18	11	4	3
Congenital MD	10	7	1	2

**Fig. 4:**
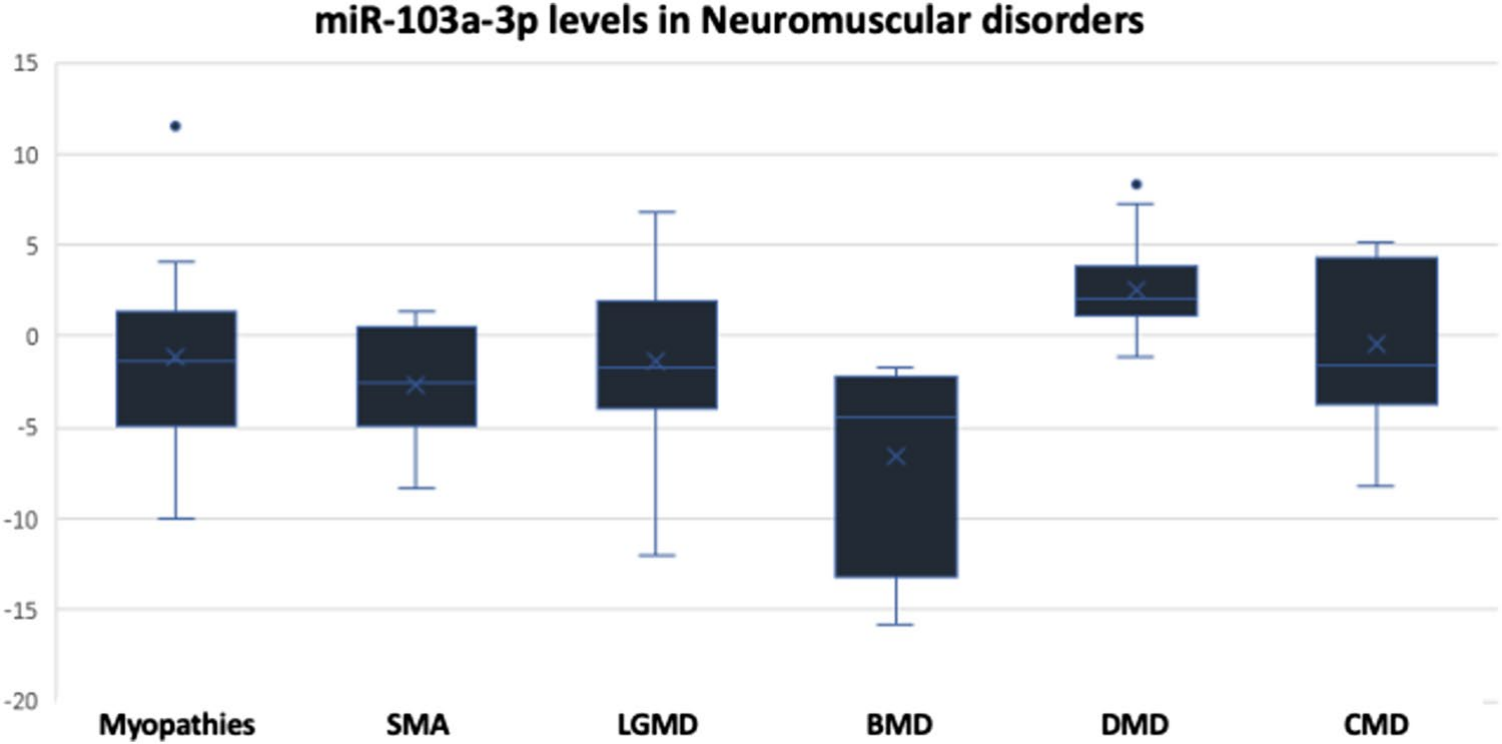
Expression levels of miR-103a-3p represented in RQ values in different neuromuscular disorders compared to the control group

When quantifying the circulating levels of miR-103a-3p using qPCR, our data revealed that BMD patients exhibited the lowest levels of miR-103a-3p as compared to the levels determined in patients with other tested diseases. It is worth to mention that the circulating levels of this miRNA species in BMD patients were significantly lower than that of the patients with DMD, and hence, it can be used to differentiate between DMD and BMD since DMD showed significant upregulation of miR-103a-3p. Regarding the serum levels in patients with LGMD, 10 patients (55.56%) had decreased levels of this miRNA species while 4 patients (22.22%) had significantly high levels and the other 4 patients (22.22%) had normal values. In case of SMA patients, 50% of the patients (9 patients out of 18) had low circulating levels and the other 50% had normal-like values. For patients with different types of myopathies, 9 patients (60%) had high levels, 4 patients (26.67%) had low levels, and the other 2 patients (13.33%) the levels were comparable to that of the control group **(**Fig. 4 and Table 4). The mean RQ values of DMD > LGMD > Congenital MDs > Myopathies > SMA > BMD. Statistical analysis showed that miR-103a-3p can be utilized to discriminate between cases with different muscular dystrophies, especially to differentiate between BMD and DMD (*p* = 0.000).

**Table 4:** Descriptive analysis showing the frequency of cases with increased or decreased levels of miR-103a-3p across different disorders:

Disease & miR-103a-3p levels	Total no	RQ > 1.5	RQ < 0.9	RQ 0.9–1.5
Myopathies	15	3	7	5
Spinal muscular atrophy	8	0	4	4
Limb-girdle muscular dystrophy	18	4	10	4
Becker muscular dystrophy	5	0	5	0
Duchenne muscular dystrophy	18	11	1	6
Congenital MD	10	4	5	1

**Fig. 5:**
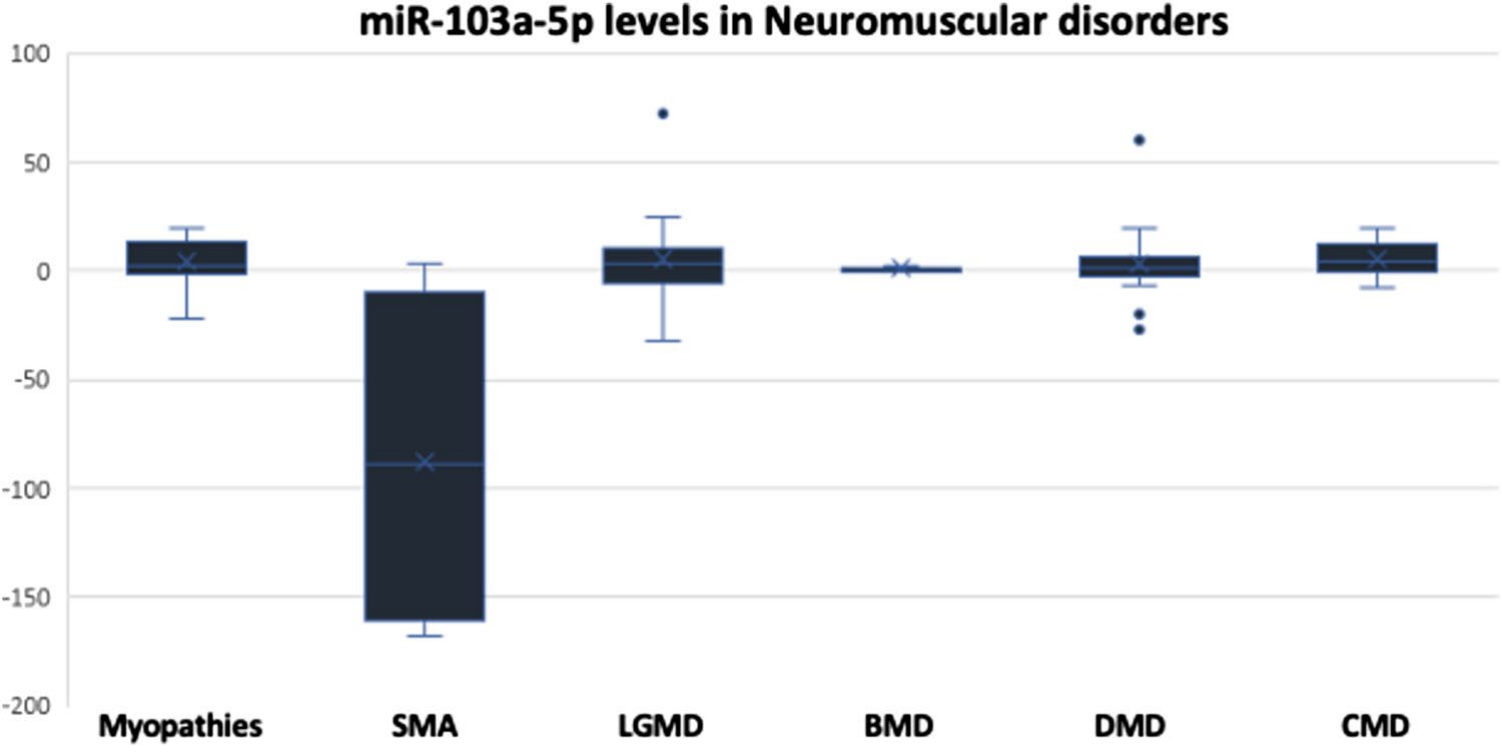
Expression levels of miR-103a-5p represented in RQ values in different neuromuscular disorders compared to the control group

We next examined the levels of miR-103a-5p in the enrolled subjects; however, there was no specific pattern for its levels except in Spinal Muscular Atrophy since it showed a highly significant downregulation in 75% of the patients (6 out of 8 patients) compared to the control groups and compared to other disorders (mean RQ = − 254.97) (Fig. 5 and Table 5). While in LGMD and DMD, 55.56% of the patients (10 patients out of 18) had high levels of miR-103a-5p, and the rest of patients in case of LGMD had higher levels of miR-103a-5p. The circulating levels of this miRNA also did not show a specific pattern in myopathies, BMD, or Congenital MDs.

**Table 5:** Descriptive analysis showing the frequency of cases with increased or decreased levels of miR-103a-5p across different disorders:

Disease & miR-103a-5p levels	Total no	RQ > 1.5	RQ < 0.9	RQ 0.9–1.5
Myopathies	15	9	4	2
Spinal muscular atrophy	8	1	6	1
Limb-girdle muscular dystrophy	18	10	8	0
Becker muscular dystrophy	5	2	0	3
Duchenne muscular dystrophy	18	10	5	3
Congenital MD	10	6	1	3

**Fig. 6:**
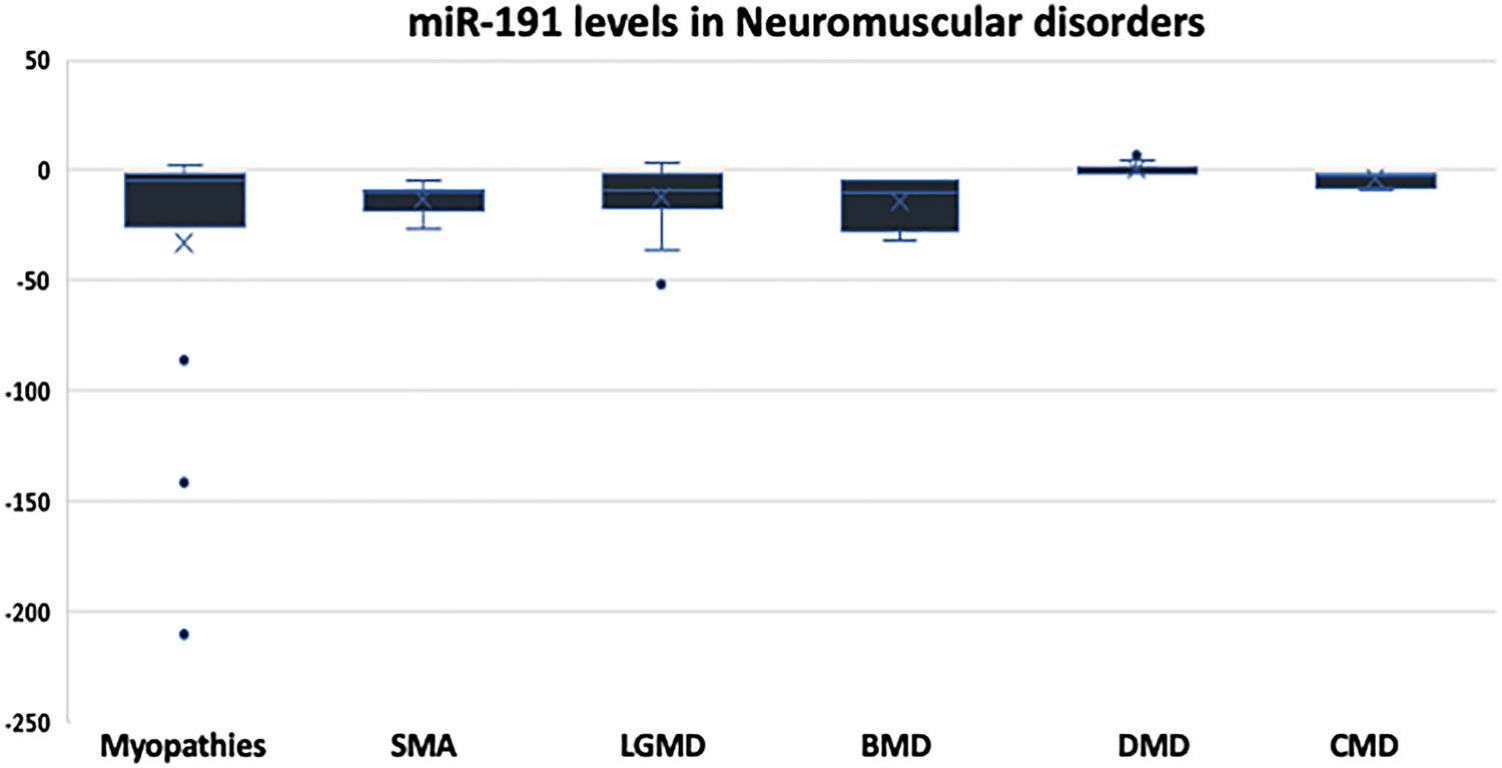
Expression levels of miR-191 represented in RQ values in different neuromuscular disorders compared to the control group

In addition, we evaluated the levels of miR-191 in the enrolled subjects, and we found that all the 8 patients of SMA as well as the 5 patients with BMD (100%, each) showed low levels of miR-191. Also, 15 patients out of 18 LGMD patients (83.33%) showed downregulation in the levels of miR-191. In contrast, 12 patients of DMD subgroup (66.67%) had control-like values, and only 4 patients (22.22%) had slightly upregulated levels and 2 patients (11.11%) showed slight downregulation. Also, 66.67% of the myopathy patients (10 patients out of 15) had low levels of miR-191 (Fig. 6 and Table 6). After analyzing the mean values, we found that miR-191 can differentiate between SMA and other disorders and can discriminate between DMD and BMD (*p* = 0.000) and discriminate between DMD and LGMD (*p* = 0.000).

**Table 6:** Descriptive analysis showing the frequency of cases with increased or decreased levels of miR-191 across different disorders:

Disease & miR-191 levels	Total no	RQ > 1.5	RQ < 0.9	RQ 0.9–1.5
Myopathies	15	3	10	2
Spinal muscular atrophy	8	0	8	0
Limb-girdle muscular dystrophy	18	3	15	0
Becker muscular dystrophy	5	0	5	0
Duchenne muscular dystrophy	18	4	2	12
Congenital MD	10	0	6	2

**Fig. 7:**
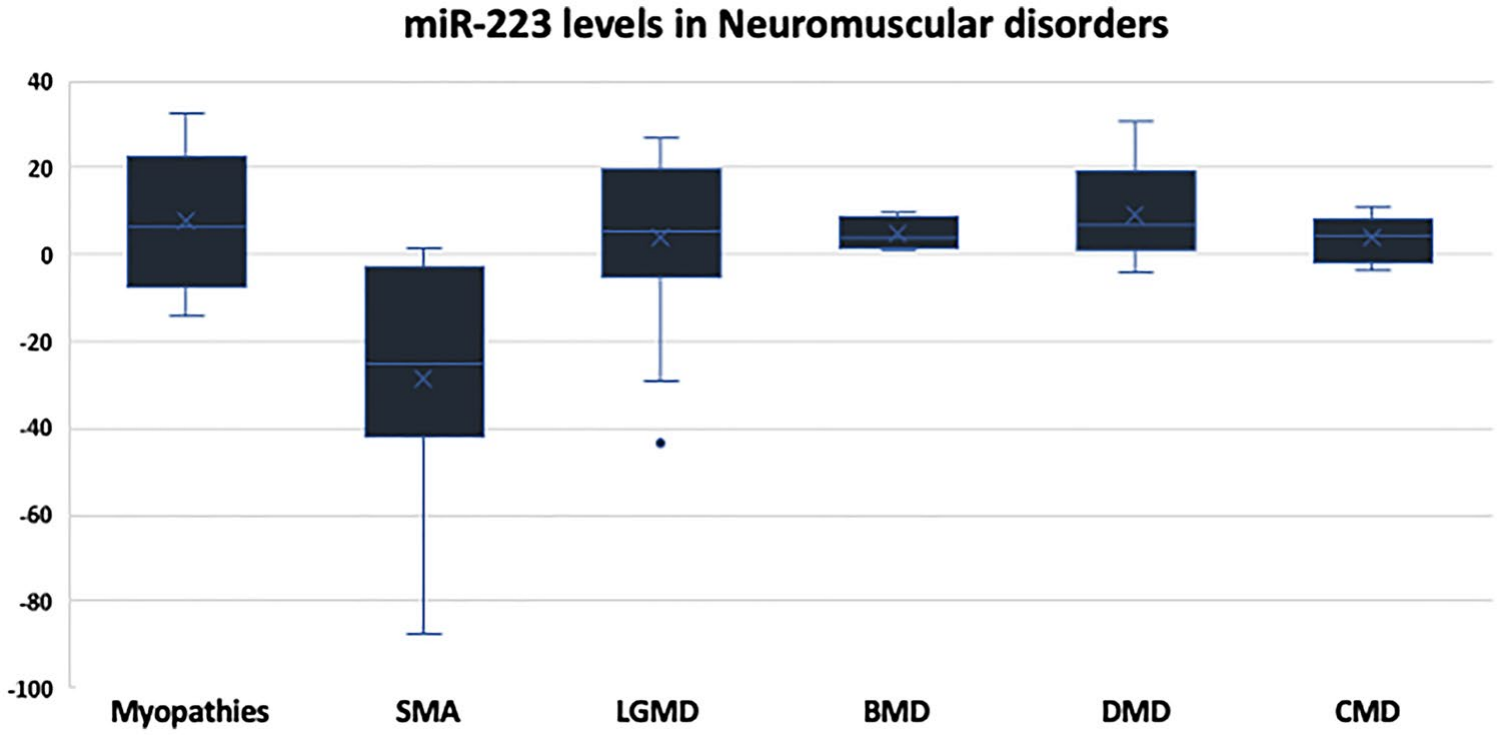
Expression levels of miR-223 represented in RQ values in different neuromuscular disorders compared to the control group

Moreover, the levels of miR-223 were determined in the sera of the enrolled subjects, and 72.22% (13 patients) of the 18 DMD patients, 60% of Congenital MDs patients and BMD (6 and 3 patients, respectively), and 55.56% of LGMD patients (10 patients) had upregulation in the levels of miR-223. However, none of the SMA patients had high levels of this miRNA species **(**Fig. 7 and Table 7). The mean RQ values in SMA (− 28.84) < congenital MDs (2.98) < BMD (4.06) < Myopathy (7.76) < LGMD (9.67) < DMD (12.77). Due to this difference between the status of miR-223 in SMA and other NMDs, we suggest that it can be used to differentiate between SMA and other disorders. However, it cannot be used to differentiate between different muscular dystrophies.

**Table 7:** Descriptive analysis showing the frequency of cases with increased or decreased levels of miR-223 across different disorders:

Disease & miR-223 levels	Total no	RQ > 1.5	RQ < 0.9	RQ 0.9–1.5
Myopathies	15	9	6	0
Spinal muscular atrophy	8	0	6	2
Limb-girdle muscular dystrophy	18	10	6	2
Becker muscular dystrophy	5	3	0	2
Duchenne muscular dystrophy	18	13	2	3
Congenital MD	10	6	4	0

**Fig. 8:**
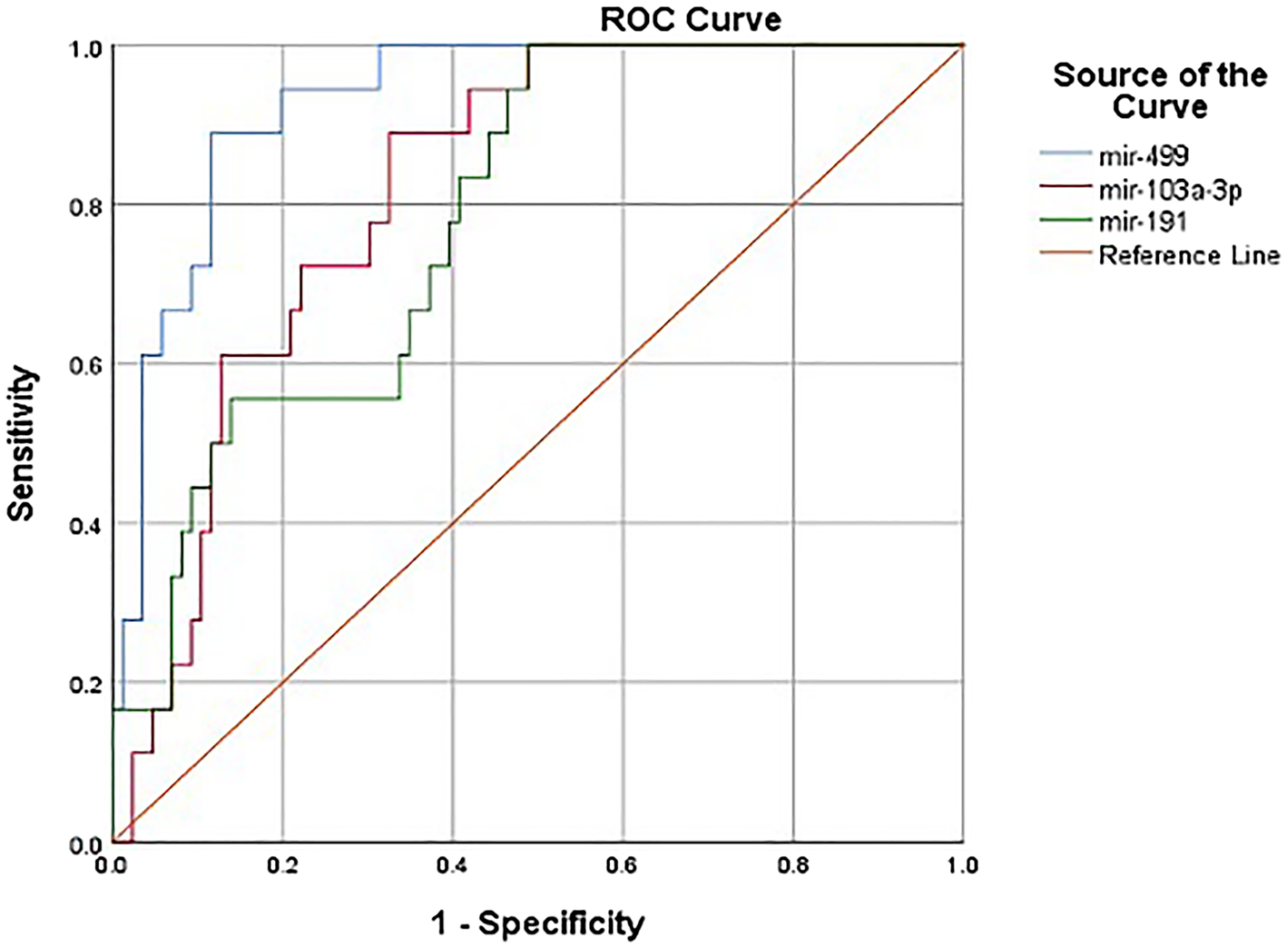
ROC analysis of circulating microRNAs level. Plotted ROC curves showed the potential of MicroRNAs (miR-499, miR-103a-3p, and miR-191-5p) to distinguish DMD from other disorders

Receiver operating characteristics curve (ROC) analysis was performed to assess the possibilities of using some or all the investigated miRNA species to differentiate DMD from other disorders (Fig. 8). The analysis revealed that miR-499 can be used as a potential biomarker to diagnose DMD and differentiate it from all other disorders (AUC = 0.931). Also, miR-103a-3p and miR-191-5p showed comparable results to miR-499 in distinguishing DMD patients from other NMD patients (AUC = 0.82 and 0.784, respectively; *p* ≤ 0.0001). However, miR-206, miR-208a, miR-223, and miR-103a-5p did not show significant difference between the tested disorders and, hence, cannot be used in the detection of the disease and distinguishing DMD from other neuromuscular Disorders (Suppl. Fig. 1). Correlations between the levels of different microRNA species are shown in Table 8.

**Table 8:** Spearman-rho correlations to test the association between the tested microRNA species in the NMD patients

	mir-499	mir-206	mir-208a	mir-103a-3p	mir-103a-5p	mir-191	mir-223
mir-499							
Correlation coefficient	1.000	.205	− .097	.089	−.122	.185	−.029
Sig. (2-tailed)		.082	.416	.452	.303	.117	.808
mir-206							
Correlation coefficient	.205	1.000	.419	.514	.404	.500	.480
Sig. (2-tailed)	.082		.000	.000	.000	.000	.000
mir-208a							
Correlation coefficient	−.097	.419	1.000	.335	.843*	.470	.808*
Sig. (2-tailed)	.416	.000		.004	.000	.000	.000
mir-103a-3p							
Correlation coefficient	.089	.514	.335	1.000	.317	.814*	.434
Sig. (2-tailed)	.452	.000	.004		.006	.000	.000
mir-103a-5p							
Correlation coefficient	− .122	.404	.843*	.317	1.000	.431	.831*
Sig. (2-tailed)	.303	.000	.000	.006		.000	.000
mir-191							
Correlation coefficient	.185	.500	.470	.814*	.431	1.000	.549
Sig. (2-tailed)	.117	.000	.000	.000	.000		.000
mir-223							
Correlation coefficient	− .029	.480	.808*	.434	.831*	.549	1.000
Sig. (2-tailed)	.808	.000	.000	.000	.000	.000	

*Correlation is significant at the 0.01 level (2-tailed)

## Discussion

MicroRNAs are a class of molecules that play an important role in the process of regulation of gene expression in the cell. The expression of the microRNAs is tissue dependent, and some microRNAs are highly expressed in the skeletal muscle cells as well as cardiomyocytes (like miR-499, miR-206 and miR-208), and thus, they play crucial roles in the regulation of the expression of muscular proteins (Sjogren et al., 2017). MicroRNAs function in maintaining the healthy status of the muscle tissue and the regeneration of the muscle cells. Dysregulation in the miRNA expression is one of the etiological factors of muscle diseases and myopathies (Perbellini et al., 2011). Some studies revealed that neuromuscular disorders including muscular dystrophies and atrophies are usually associated with the aberrant expression of the microRNAs and the abnormal localization of some species of microRNAs (Coenen-Stass et al., 2017). Also, muscle pathologies affect the levels of some microRNA species in various body fluids like blood and urine. Duchenne muscular dystrophy is one of the most aggressive forms of muscle dystrophies, and some studies highlighted the fact that the levels of some muscle-specific microRNAs are altered in tested muscle biopsies or serum samples compared to normal samples (Cacchiarelli et al., 2010; Giordani et al., 2014; Hu et al., 2014; Meng & Lan, 2019; Mousa et al., 2020). MicroRNA levels also differ in chronic muscle diseases that are characterized by muscle degeneration, which occurs as a secondary event due to the degeneration of the motor neurons) like ALS (Hoye et al., 2017; Ridler, 2018; Rizzuti et al., 2018). In addition, patients with myopathies (Ex: Laminopathies) were found to have dysregulation in a panel of microRNAs that found to be essential in myoblast proliferation (Sylvius et al., 2011).

In this study, we investigated the levels of seven miRNA species in neuromuscular disorders that have comparable symptoms. The first tested species was miR-499, a well-known muscle-specific miRNA that is usually associated with mitochondrial function in muscles and the contractility of the muscle fibers (Wang et al., 2019; Wu et al., 2019). In a previous study, we found that miRNA-499 levels in DMD patients were extremely upregulated in the plasma samples compared to the control subjects. Interestingly, in the current study, quantitatively, the circulating levels of miR-499 were found to be the highest in DMD patients, followed by LGMD, then SMA patients and were significantly lower in BMD and myopathies. In fact, this pattern could be easily explained in the light of the extent of muscle damage in each disease and the disease etiology. Obviously, DMD is the most aggressive among all NMDs due to the absence of an important muscular protein “dystrophin” since the increased Ca^2+^ influx into the myocytes, due to the comprised integrity of the sarcolemma, consequently leads to Ca^2+^ overload inside the mitochondria that will end up with mitochondrial dysfunction and activation of apoptotic mechanisms that causes accelerated cellular death (Rybalka et al., 2014). The damage in case of the DMD is enormous, it is always associated with mitochondrial dysfunction and the dystrophy of muscle fibers through necrosis. Upon being severely injured, the skeletal muscles tend to release processed miRNAs into the circulation, and hence, the levels will be increase significantly. The levels of miR-499 were also significantly upregulated in LGMD patients; however, the severity of the disease differs from case to case. Some LGMD patients have early onset of the symptoms; however, other cases show the signs in their adulthood. In fact, dystrophinopathy is the most common form of MD, and thus, any patient presenting Limb-Girdle symptoms should be suspected also to have DMD or BMD (Iyadurai & Kissel, 2016). The LGMD patients suffer from mild to severe muscle atrophy that starts from the proximal muscles of hips and shoulders that extends to reach the distal muscles as well (Wang et al., 2018). The levels of miRNA-499 were also upregulated in LGMD patients since the defect occurs in muscular proteins like Dysferlin, Calpain, Sarcoglycan, Titin, and other proteins which are responsible for the onset of LGMD and cause muscle injuries. In fact, SMA patients also suffer from elevation in miR-499 levels; however, this elevation is lower than MDs, and this might be attributed to the fact that SMA mainly occurs in the motor neurons not the muscle tissue. Also, SMA type 1 is the most severe form of the disease and occurs within the first few months in life; however, type III and type IV are not as severe as type I (Arnold et al., 2015). The two main hallmarks of SMA are the motor neuron death and the denervation of the muscles, but the muscular proteins are intact and normal (Simone et al., 2016). The muscles usually reach the atrophy status due to the disruption of the signaling pathways between spinal cord and muscles. The atrophy that occurs in the SMA muscles is a secondary of the innervation, and many studies showed that physiotherapy can maintain the integrity of the muscles even if it is not capable of reversing the atrophied muscles (Arnold et al., 2014). This means that the damage that occurs in case of SMA patients is less than that occurs in MDs, and this justifies the difference of microRNAs levels between different cases.

Next, we examined the levels of miR-206 in the selected disorders. MiR-206 is expressed in the skeletal muscles as well as in Spinal Motor Neurons, and it plays an important role in the generation of neuromuscular junctions after injury (Hawley et al., 2017). Previously, we found that miRNA was downregulated in DMD patients, and similar results were obtained in the current investigations. However, the levels of miR-206 were significantly downregulated in SMA compared to other disorders. MiR-206 is one of the important modulators in skeletal muscles, and it was previously linked the upregulation of miR-206 to some diseases like Amyotrophic lateral sclerosis (Pietro et al., 2017), DMD, and SMA (Valsecchi, 2020). In other study by Bulaklak et al., they discovered that miR-206 downregulation could improve the motor function and the muscle pathology since this downregulation allows the expression of beneficial genes (Bulaklak et al., 2018). In another recent study that was published in 2020, they found that miR-206 reduces the severity of the neuronal degeneration accompanied with SMA, and hence, it plays a neuroprotective role, and this explains why the levels of miR-206 were significantly downregulated in the plasma of SMA patients (Valsecchi, 2020).

In addition, we examined the levels of miR-208a which is related to the cardiac tissue and currently used to reflect the status of the heart tissue and to indicate if there is a cardiomyopathy or not (Zhou et al., 2018). In DMD patients, 11 patients had elevated level of miR-208a. In fact, miR-208a is essential for conducting a proper cardiac conduction and to maintain the normal expression of heart-specific transcription factor (Xiao et al., 2014). The upregulation of miR-208a is usually indicating the presence of cardiac hypertrophy (Shyu et al., 2015), which is a common symptom in the MDs. Regarding SMA, there is no relationship between SMA and heart diseases; however, some studies reported that arrythmia and cardiac defects are common feature of SMA (Wijngaarde et al., 2017). In our study, SMA patients had low levels of miR-208a compared to the control subjects which indicates that SMA patients show no signs of myocardial infarction.

Furthermore, we investigated the levels of miR-103a-3p which indicate the presence of inflammatory response. In our previous study, miR-103a-3p was significantly upregulated in 93% of the patients. In the current study, DMD patients showed the highest mean value of miR-103a-3p among all groups, and similar results were obtained in case of miR-103a-5p. However, BMD had much lower levels of miR-103a-3p compared to DMD patients because the systemic inflammation is higher in DMD patients and lower in the milder form BMD with the better muscle function.

In conclusion, microRNAs dysregulation plays a prominent role in the muscular system and its maintenance, and its aberration can adversely affect the muscle cells leading to muscle disorders. Hence, Deciphering the exact mechanism by which microRNA expression plays role in muscle cell differentiation and regeneration can help in better understanding the role of these microRNAs in different muscle disorders and can pave the way towards generating targeted therapies. In our study, the sample size of some of the investigated diseases, namely BMD and SMA, was relatively small, a condition that hindered drawing solid conclusions regarding identifying disease-specific miRNAs for these diseases. However, our results highlighted the significance of identified miRNA species that showed correlations with the occurrence of those muscular disorders in BMD and SMA patients. The scope of this study will be extended further in our future work by including larger cohorts for the investigated diseases to validate our findings.

### Supplementary Information

Below is the link to the electronic supplementary material.

Supplementary file1 (DOCX 89 KB)

## Data Availability

No data associated in the manuscript.

## Abbreviations

MD: Muscular dystrophy
CMD: Congenital muscular dystrophy
NMD: Neuromuscular disorders
DMD: Duchenne muscular dystrophy
BMD: Becker muscular dystrophy
MD: Myotonic dystrophy
LGMD: Limb-girdle muscular dystrophy
CK: Creatine kinase
AR: Autosomal recessive
AD: Autosomal dominant
SMA: Spinal muscular atrophy
