# Supplementary material for: MicroRNAs as a Tool for Differential Diagnosis of Neuromuscular Disorders
Source: Neuromolecular Med. 2023 Oct 19;25(4):603–15. doi: 10.1007/s12017-023-08763-0 (PMC10721695; doi:10.1007/s12017-023-08763-0)
Supplement: Supplementary file 1 — Supplementary file1 (DOCX 89 KB) [file 12017_2023_8763_MOESM1_ESM.docx]

**MicroRNAs as a tool for Differential Diagnosis of Neuromuscular Disorders**

Nahla O. Mousa ^1^, Ahmed Abdellatif *^2^, Nagia Fahmy ^3^, Hassan El-Fawal ^4^, Ahmed Osman *^5, 6^

***Corresponding Authors:**

**Ahmed Abdellatif**, [ahmed.abdellatif@aucegypt.edu](mailto:ahmed.abdellatif@aucegypt.edu), Biology Department, School of Sciences & Engineering, The American University in Cairo, [School of Sciences and Engineering](https://www.researchgate.net/institution/The_American_University_in_Cairo/department/School_of_Sciences_and_Engineering), 11835, Cairo, Egypt.

**Ahmed Osman**, [ahmed.osman@ejust.edu.eg](mailto:ahmed.osman@ejust.edu.eg) Biotechnology Department, Basic and Applied sciences Institute, Egypt-Japan University of Science and Technology, Borg Al Arab, 21934, Egypt.

**Supplementary Figure (1):** ROC analysis of circulating microRNAs level. Plotted ROC curves showed the potential of MicroRNAs (miR-206, miR-208a, miR-103a-5p and miR-223) to distinguish DMD from other disorders.


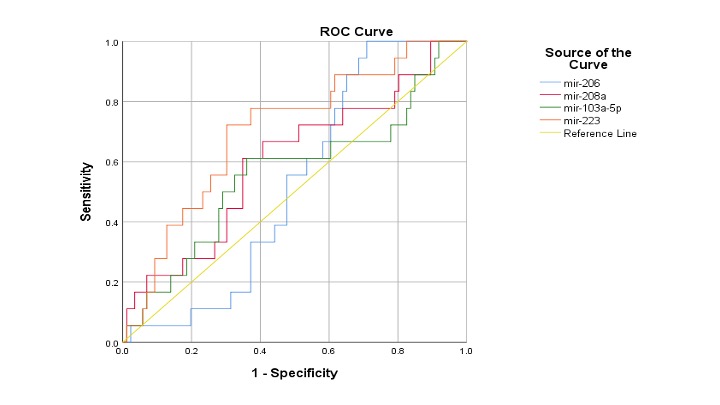


**Supplementary Table (1):** Descriptive analysis showing the mean, standard deviation, minimum and maximum levels of miR-499 RQ values in the selected disorders:

| Disease | Mean | N | Std. Deviation | Minimum | Maximum |
| --- | --- | --- | --- | --- | --- |
| Myopathy | 22.101237485260626 | 15 | 69.132266696231160 | -28.933239558775043 | 238.721542455570000 |
| Spinal muscular atrophy | 102.819672385215140 | 8 | 182.407358168962870 | 12.987953420000000 | 543.526088734440000 |
| Limb-Girdle MD | 144.777806201489740 | 18 | 200.676819879277420 | -31.146539973063668 | 664.361047365840000 |
| Becker MD | 98.767173781638090 | 5 | 89.785397722199760 | 34.568900230000000 | 242.022941888190450 |
| Duchenne MD | 418.151152953760060 | 18 | 240.572295723732850 | 24.238878800000000 | 850.420021374500200 |
| Congenital MD | 83.136121257502670 | 10 | 107.622827343001730 | -3.206501317597577 | 297.965192806947240 |

**Supplementary Table (2):** Pairwise comparisons of the levels of miR-499 across the selected disorders using Kruskal-Wallis test

| Sample 1-Sample 2 | Sig. | Adj. Sig.^a^ |
| --- | --- | --- |
| **Myopathy-Control** | **.000** | **.000** |
| Myopathy-Limb-Girdle MD | **.037** | 1.000 |
| Myopathy-Congenital MD | **.011** | .321 |
| Myopathy-Spinal muscular atrophy | **.006** | .177 |
| Myopathy-Becker MD | **.009** | .249 |
| **Myopathy-Duchenne MD** | **.000** | **.000** |
| **Control-Limb-Girdle MD** | **.000** | **.000** |
| **Control-Congenital MD** | **.000** | **.000** |
| **Control-Spinal muscular atrophy** | **.000** | **.000** |
| **Control-Becker MD** | **.000** | **.000** |
| **Control-Duchenne MD** | **.000** | **.000** |
| Limb-Girdle MD-Congenital MD | .443 | 1.000 |
| Limb-Girdle MD-Spinal muscular atrophy | .274 | 1.000 |
| Limb-Girdle MD-Becker MD | .219 | 1.000 |
| **Limb-Girdle MD-Duchenne MD** | **.000** | **.004** |
| Congenital MD-Spinal muscular atrophy | .732 | 1.000 |
| Congenital MD-Becker MD | .561 | 1.000 |
| Congenital MD-Duchenne MD | **.015** | .414 |
| Spinal muscular atrophy-Becker MD | .784 | 1.000 |
| Spinal muscular atrophy-Duchenne MD | .060 | 1.000 |
| Becker MD-Duchenne MD | .204 | 1.000 |

**Supplementary Table (3):** Descriptive analysis showing the mean, standard deviation, minimum and maximum levels of miR-206 RQ values in the selected disorders:

| Disease | Mean | N | Std. Deviation | Minimum | Maximum |
| --- | --- | --- | --- | --- | --- |
| Myopathy | -135.211345572058630 | 15 | 181.368435038110330 | -576.029944160670900 | -6.462524646776497 |
| Spinal muscular atrophy | -1222.449446735413900 | 8 | 1743.498272129843600 | -4803.931952053772000 | -6.983742448182482 |
| Limb-Girdle MD | -427.198295924491840 | 18 | 747.106189576685000 | -2134.969878202614400 | 1.347233576865691 |
| Becker MD | -133.042578975464520 | 5 | 184.256663497661800 | -458.252836315122750 | -31.124958317193077 |
| Duchenne MD | -21.302336345666127 | 18 | 21.617346382972560 | -74.288089806574620 | 1.214194884395045 |
| Congenital MD | -40.848545671199820 | 10 | 55.770773153339846 | -187.402969081039630 | -5.290064974958551 |

**Supplementary Table (4):** Pairwise comparisons of the levels of miR-206 across the selected disorders using Kruskal-Wallis test.

| Sample 1-Sample 2 | Sig. | Adj. Sig.^a^ |
| --- | --- | --- |
| Spinal muscular atrophy-Becker MD | .609 | 1.000 |
| Spinal muscular atrophy-Myopathy | .385 | 1.000 |
| Spinal muscular atrophy-Limb-Girdle MD | .238 | 1.000 |
| Spinal muscular atrophy-Congenital MD | .069 | 1.000 |
| **Spinal muscular atrophy-Duchenne MD** | **.007** | **.191** |
| **Spinal muscular atrophy-Control** | **.000** | **.000** |
| Becker MD-Myopathy | .864 | 1.000 |
| Becker MD-Limb-Girdle MD | .679 | 1.000 |
| Becker MD-Congenital MD | .298 | 1.000 |
| Becker MD-Duchenne MD | .090 | 1.000 |
| **Becker MD-Control** | **.000** | **.002** |
| Myopathy-Limb-Girdle MD | .730 | 1.000 |
| Myopathy-Congenital MD | .238 | 1.000 |
| Myopathy-Duchenne MD | **.028** | .779 |
| **Myopathy-Control** | **.000** | **.000** |
| Limb-Girdle MD-Congenital MD | .360 | 1.000 |
| Limb-Girdle MD-Duchenne MD | .052 | 1.000 |
| **Limb-Girdle MD-Control** | **.000** | **.000** |
| Congenital MD-Duchenne MD | .466 | 1.000 |
| **Congenital MD-Control** | **.000** | **.006** |
| **Duchenne MD-Control** | **.000** | **.009** |

**Supplementary Table (5):** Descriptive analysis showing the mean, standard deviation, minimum and maximum levels of miR-206 RQ values in the selected disorders:

| Disease | Mean | N | Std. Deviation | Minimum | Maximum |
| --- | --- | --- | --- | --- | --- |
| Myopathy | 14.408030406052323 | 15 | 21.568371480338094 | -11.352986849603060 | 66.949436146721880 |
| Spinal muscular atrophy | -228.738118393170050 | 8 | 423.507205272146560 | -1256.330216707893400 | 5.407643332112508 |
| Limb-Girdle MD | -9.634911257052874 | 18 | 112.034562448109330 | -438.064653149491850 | 120.676420597534830 |
| Becker MD | -3.037722723375316 | 5 | 15.732264534668674 | -30.803022179256068 | 7.037192607379486 |
| Duchenne MD | 16.281026295040120 | 18 | 35.928683874611544 | -14.876879081810593 | 120.676420597534500 |
| Congenital MD | 5.027165797831104 | 10 | 7.455739421236163 | -8.196454584262417 | 19.359905427508654 |

**Supplementary Table (6):** Pairwise comparisons of the levels of miR-208a across the selected disorders using Kruskal-Wallis test.

| Sample 1-Sample 2 | Sig. | Adj. Sig.^a^ |
| --- | --- | --- |
| Spinal muscular atrophy-Control | .089 | 1.000 |
| Spinal muscular atrophy-Becker MD | **.031** | .855 |
| **Spinal muscular atrophy-Limb-Girdle MD** | **.004** | **.102** |
| **Spinal muscular atrophy-Duchenne MD** | **.001** | **.036** |
| **Spinal muscular atrophy-Congenital MD** | **.001** | **.040** |
| **Spinal muscular atrophy-Myopathy** | **.000** | **.009** |
| Control-Becker MD | .250 | 1.000 |
| Control-Limb-Girdle MD | .062 | 1.000 |
| Control-Duchenne MD | **.020** | .573 |
| Control-Congenital MD | **.022** | .625 |
| **Control-Myopathy** | **.005** | **.130** |
| Becker MD-Limb-Girdle MD | .997 | 1.000 |
| Becker MD-Duchenne MD | .789 | 1.000 |
| Becker MD-Congenital MD | .611 | 1.000 |
| Becker MD-Myopathy | .511 | 1.000 |
| Limb-Girdle MD-Duchenne MD | .689 | 1.000 |
| Limb-Girdle MD-Congenital MD | .483 | 1.000 |
| Limb-Girdle MD-Myopathy | .334 | 1.000 |
| Duchenne MD-Congenital MD | .716 | 1.000 |
| Duchenne MD-Myopathy | .559 | 1.000 |
| Congenital MD-Myopathy | .882 | 1.000 |

**Supplementary Table (7):** Descriptive analysis showing the mean, standard deviation, minimum and maximum levels of miR-103a-3p RQ values in the selected disorders:

| Disease | Mean | N | Std. Deviation | Minimum | Maximum |
| --- | --- | --- | --- | --- | --- |
| Control | .185997185024221 | 30 | 1.169799159153553 | -1.248330548901610 | 1.494849248634940 |
| Myopathy | -1.172682791704658 | 15 | 5.278861434333867 | -10.038468112499867 | 11.531433788653565 |
| Spinal muscular atrophy | -2.700624821422667 | 8 | 3.314809227944563 | -8.400753510626588 | 1.329220830000000 |
| Limb-Girdle MD | 1.420337822972559 | 18 | 14.519275478752148 | -11.979534965417017 | 56.395181638188360 |
| Becker MD | -6.364339014890526 | 5 | 5.488479386933305 | -15.807091154248264 | -1.708227352690600 |
| Duchenne MD | 2.562634064883951 | 18 | 2.469379032930358 | -1.081100017834264 | 8.267779999193404 |
| Congenital MD | -.516332899528486 | 10 | 4.398340755284752 | -8.239175653399200 | 5.089415578385902 |
| Total | .010511009763992 | 104 | 6.917310030262382 | -15.807091154248264 | 56.395181638188360 |

**Supplementary Table (8):** Pairwise comparisons of the levels of miR-103a-3p across the selected disorders using Kruskal-Wallis test.

| Sample 1-Sample 2 | Sig. | Adj. Sig.^a^ |
| --- | --- | --- |
| Becker MD-Spinal muscular atrophy | .243 | 1.000 |
| Becker MD-Limb-Girdle MD | **.053** | 1.000 |
| Becker MD-Myopathy | **.041** | 1.000 |
| Becker MD-Congenital MD | **.028** | .772 |
| **Becker MD-Control** | **.003** | **.082** |
| **Becker MD-Duchenne MD** | **.000** | **.000** |
| Spinal muscular atrophy-Limb-Girdle MD | .463 | 1.000 |
| Spinal muscular atrophy-Myopathy | .372 | 1.000 |
| Spinal muscular atrophy-Congenital MD | .254 | 1.000 |
| Spinal muscular atrophy-Control | **.053** | 1.000 |
| **Spinal muscular atrophy-Duchenne MD** | **.000** | **.009** |
| Limb-Girdle MD-Myopathy | .821 | 1.000 |
| Limb-Girdle MD-Congenital MD | .561 | 1.000 |
| Limb-Girdle MD-Control | .123 | 1.000 |
| **Limb-Girdle MD-Duchenne MD** | **.000** | **.007** |
| Myopathy-Congenital MD | .713 | 1.000 |
| Myopathy-Control | .229 | 1.000 |
| **Myopathy-Duchenne MD** | **.001** | **.032** |
| Congenital MD-Control | .529 | 1.000 |
| Congenital MD-Duchenne MD | **.012** | .343 |
| Control-Duchenne MD | **.011** | .308 |

**Supplementary Table (9):** Descriptive analysis showing the mean, standard deviation, minimum and maximum levels of miR-103a-5p RQ values in the selected disorders:

| Disease | Mean | N | Std. Deviation | Minimum | Maximum |
| --- | --- | --- | --- | --- | --- |
| Control | .210097537722337 | 30 | 1.152873999141494 | -1.484523570629050 | 1.414213562373094 |
| Myopathy | 4.049711974844396 | 15 | 11.527919833676874 | -21.856644108070370 | 19.106746370000000 |
| Spinal muscular atrophy | -254.972084626332100 | 8 | 503.203902983807440 | -1488.867857735997000 | 3.052518417921117 |
| Limb-Girdle MD | -10.177854439917867 | 18 | 77.489993894527980 | -310.833890405131500 | 72.504568658931100 |
| Becker MD | .471121392001832 | 5 | 1.399846622277392 | -1.035895920333894 | 1.734576643000000 |
| Duchenne MD | 3.509071333009265 | 18 | 18.007563670393758 | -27.284316536574604 | 60.129455949696755 |
| Congenital MD | 4.689250234789404 | 10 | 8.214025783425907 | -8.224910613248529 | 19.698310613518615 |
| Total | -19.649212094930530 | 104 | 151.532398009837800 | -1488.867857735997000 | 72.504568658931100 |

**Supplementary Table (10):** Pairwise comparisons of the levels of miR-103a-5p across the selected disorders using Kruskal-Wallis test.

| Sample 1-Sample 2 | Sig. | Adj. Sig.^a^ |
| --- | --- | --- |
| Spinal muscular atrophy-Control | **.041** | 1.000 |
| Spinal muscular atrophy-Becker MD | .063 | 1.000 |
| **Spinal muscular atrophy-Limb-Girdle MD** | **.006** | **.168** |
| **Spinal muscular atrophy-Duchenne MD** | **.004** | **.120** |
| **Spinal muscular atrophy-Myopathy** | **.002** | **.047** |
| **Spinal muscular atrophy-Congenital MD** | **.001** | **.033** |
| Control-Becker MD | .610 | 1.000 |
| Control-Limb-Girdle MD | .236 | 1.000 |
| Control-Duchenne MD | .180 | 1.000 |
| Control-Myopathy | .076 | 1.000 |
| Control-Congenital MD | **.047** | 1.000 |
| Becker MD-Limb-Girdle MD | .833 | 1.000 |
| Becker MD-Duchenne MD | .762 | 1.000 |
| Becker MD-Myopathy | .543 | 1.000 |
| Becker MD-Congenital MD | .383 | 1.000 |
| Limb-Girdle MD-Duchenne MD | .890 | 1.000 |
| Limb-Girdle MD-Myopathy | .554 | 1.000 |
| Limb-Girdle MD-Congenital MD | .347 | 1.000 |
| Duchenne MD-Myopathy | .645 | 1.000 |
| Duchenne MD-Congenital MD | .411 | 1.000 |
| Myopathy-Congenital MD | .689 | 1.000 |

**Supplementary Table (11):** Descriptive analysis showing the mean, standard deviation, minimum and maximum levels of miR-191 RQ values in the selected disorders:

| Disease | Mean | N | Std. Deviation | Minimum | Maximum |
| --- | --- | --- | --- | --- | --- |
| Control | .412001987519419 | 30 | 1.119456394758872 | -1.494849248634940 | 1.453972517320311 |
| Myopathy | -33.030491150880670 | 15 | 63.356019635690650 | -210.363162549298580 | 2.660953616000000 |
| Spinal muscular atrophy | -12.887666723858814 | 8 | 7.109256826571708 | -26.717048608250160 | -5.230516608082788 |
| Limb-Girdle MD | -11.944777634887949 | 18 | 14.994754345925791 | -51.660868768304940 | 3.552907183369620 |
| Becker MD | -12.262792190304491 | 5 | 11.519587158971875 | -31.800994986321772 | -4.375482273860254 |
| Duchenne MD | .785813389581877 | 18 | 2.384869630338356 | -1.778917987075796 | 6.448662651943737 |
| Congenital MD | -4.027451843289066 | 10 | 3.008562808936567 | -9.259921735921000 | -1.133669412778424 |
| Total | -8.544696896427121 | 104 | 26.933287129049320 | -210.363162549298580 | 6.448662651943737 |

**Supplementary Table (12):** Pairwise comparisons of the levels of miR-191 across the selected disorders using Kruskal-Wallis test.

| **Sample 1-Sample 2** | **Sig.** | **Adj. Sig.^a^** |
| --- | --- | --- |
| Spinal muscular atrophy-Becker MD | .801 | 1.000 |
| Spinal muscular atrophy-Limb-Girdle MD | .185 | 1.000 |
| Spinal muscular atrophy-Congenital MD | .172 | 1.000 |
| Spinal muscular atrophy-Myopathy | .099 | 1.000 |
| **Spinal muscular atrophy-Control** | **.000** | **.000** |
| **Spinal muscular atrophy-Duchenne MD** | **.000** | **.000** |
| Becker MD-Limb-Girdle MD | .406 | 1.000 |
| Becker MD-Congenital MD | .358 | 1.000 |
| Becker MD-Myopathy | .262 | 1.000 |
| **Becker MD-Control** | **.001** | **.021** |
| **Becker MD-Duchenne MD** | **.000** | **.012** |
| Limb-Girdle MD-Congenital MD | .831 | 1.000 |
| Limb-Girdle MD-Myopathy | .649 | 1.000 |
| **Limb-Girdle MD-Control** | **.000** | **.001** |
| **Limb-Girdle MD-Duchenne MD** | **.000** | **.001** |
| Congenital MD-Myopathy | .854 | 1.000 |
| **Congenital MD-Control** | **.002** | **.058** |
| **Congenital MD-Duchenne MD** | **.001** | **.034** |
| **Myopathy-Control** | **.001** | **.026** |
| **Myopathy-Duchenne MD** | **.001** | **.016** |
| Control-Duchenne MD | .608 | 1.000 |

**Supplementary Table (13):** Descriptive analysis showing the mean, standard deviation, minimum and maximum levels of miR-223 RQ values in the selected disorders:

| Disease | Mean | N | Std. Deviation | Minimum | Maximum |
| --- | --- | --- | --- | --- | --- |
| Control | .073567882169125 | 30 | 1.246675602561049 | -1.580082623726750 | 1.569168195793502 |
| Myopathy | 7.768271424558402 | 15 | 15.709795822886020 | -14.417992570494935 | 32.401753835215770 |
| Spinal muscular atrophy | -28.846171534165585 | 8 | 28.705270375697523 | -87.547859449759330 | 1.422077410587273 |
| Limb-Girdle MD | 9.678427823570757 | 18 | 34.500317142660910 | -43.471561105566150 | 126.939741534501740 |
| Becker MD | 4.063726206690349 | 5 | 3.717284007163125 | 1.212512819061738 | 9.474394764000000 |
| Duchenne MD | 12.776924747286104 | 18 | 22.181100481347965 | -4.005549022845337 | 93.571676697601920 |
| Congenital MD | 2.983395221887517 | 10 | 5.381360785396329 | -3.635112465794078 | 11.065525032087711 |
| Total | 3.291448702768032 | 104 | 21.903222649494960 | -87.547859449759330 | 126.939741534501740 |

**Supplementary Table (14):** Pairwise comparisons of the levels of miR-223 across the selected disorders using Kruskal-Wallis test.

| Sample 1-Sample 2 | Sig. | Adj. Sig.^a^ |
| --- | --- | --- |
| Spinal muscular atrophy-Control | **.040** | 1.000 |
| Spinal muscular atrophy-Congenital MD | **.013** | .375 |
| **Spinal muscular atrophy-Limb-Girdle MD** | **.002** | **.061** |
| **Spinal muscular atrophy-Myopathy** | **.002** | **.047** |
| **Spinal muscular atrophy-Becker MD** | **.004** | **.106** |
| **Spinal muscular atrophy-Duchenne MD** | **.000** | **.001** |
| Control-Congenital MD | .328 | 1.000 |
| Control-Limb-Girdle MD | .103 | 1.000 |
| Control-Myopathy | .078 | 1.000 |
| Control-Becker MD | .084 | 1.000 |
| **Control-Duchenne MD** | **.002** | **.063** |
| Congenital MD-Limb-Girdle MD | .744 | 1.000 |
| Congenital MD-Myopathy | .622 | 1.000 |
| Congenital MD-Becker MD | .383 | 1.000 |
| Congenital MD-Duchenne MD | .161 | 1.000 |
| Limb-Girdle MD-Myopathy | .836 | 1.000 |
| Limb-Girdle MD-Becker MD | .490 | 1.000 |
| Limb-Girdle MD-Duchenne MD | .203 | 1.000 |
| Myopathy-Becker MD | .593 | 1.000 |
| Myopathy-Duchenne MD | .314 | 1.000 |
| Becker MD-Duchenne MD | .881 | 1.000 |
